# LKB1 suppression promotes cardiomyocyte regeneration via LKB1-AMPK-YAP axis

**DOI:** 10.17305/bjbms.2021.7225

**Published:** 2022-04-29

**Authors:** Shuang Qu, Qiao Liao, Cheng Yu, Yue Chen, Hao Luo, Xuewei Xia, Duofen He, Zaicheng Xu, Pedro A. Jose, Zhuxin Li, Wei Eric Wang, Qing Rex Lyu, Chunyu Zeng

**Affiliations:** 1 Department of Cardiology, Daping Hospital, The Third Military Medical University, Chongqing, China; 2 Chongqing Key Laboratory for Hypertension Research, Chongqing Cardiovascular Clinical Research Center, Chongqing Institute of Cardiology, Chongqing, China; 3 Division of Renal Diseases and Hypertension, Departments of Medicine and Pharmacology/Physiology, The George Washington University School of Medicine and Health Sciences, Washington, DC, USA; 4 Biomedical and Health Institute, Chongqing Institute of Green and Intelligence Technology, Chinese Academy of Sciences, Chongqing, China; 5 Cardiovascular Research Center of Chongqing College, University of Chinese Academy of Sciences, Chongqing, China; 6 State Key Laboratory of Trauma, Burns and Combined Injury, Daping Hospital, The Third Military Medical University, Chongqing, China

**Keywords:** LKB1, cardiomyocyte proliferation, AMPK, YAP

## Abstract

The regenerative potential of cardiomyocytes in adult mammals is limited. The previous studies reported that cardiomyocyte proliferation is suppressed by AMP-activated protein kinase (AMPK). The role of liver kinase B1 (LKB1), as the major upstream kinase for AMPK, on cardiomyocyte proliferation is unclear. In this study, we found that the LKB1 levels rapidly increased after birth. With loss- and gain-of-function study, our data demonstrated that LKB1 levels negatively correlate with cardiomyocyte proliferation. We next identified Yes-associated protein (YAP) as the downstream effector of LKB1 using high-throughput RNA sequencing. Our results also demonstrated that AMPK plays an essential role in *Lkb1* knockdown-induced cardiomyocyte proliferation. Importantly, deactivated AMPK abolished the LKB1-mediated regulation of YAP nuclear translocation and cardiomyocyte proliferation. Thus, our findings suggested the role of LKB1-AMPK-YAP axis during cardiomyocyte proliferation, which could be used as a potential target for inducing cardiac regeneration after injury.

## INTRODUCTION

As terminally differentiated cells, cardiomyocytes play an indispensable role in driving circulation by pumping blood through rhythmic contraction [1]. It is well recognized that the adult mammalian heart possesses minimal self-renewal potential that cannot compensate for the loss of cardiomyocytes post-injury, such as myocardial infarction, which results in compromised heart function and eventually heart failure [2]. Cardiomyocytes withdraw from the cell cycle shortly after birth and shut off cell cycle-related protein synthesis, such as cyclins [3]. After birth, cardiomyocytes undergo a major energy metabolic transition from anaerobic glycolysis to fatty acid oxidation [4], suggesting the vital role of metabolic transition in cardiomyocyte proliferation [5].

Cardiomyocytes, which are high energy-demand cells, are regulated by the metabolic sensor, AMP-activated protein kinase (AMPK) [6]. AMPK is a critical regulator of cellular energy homeostasis. It inhibits anabolic metabolism when AMP/ATP ratio is increased by reducing energy-consuming processes, such as protein synthesis [7]. The adult cardiomyocyte expresses abundant AMPK. The previous studies demonstrated that activated AMPK stimulates oxidative fatty acid metabolism and increases glucose transport and glycolysis to produce more energy [8,9]. AMPK strongly suppresses cell proliferation by inhibiting the mTOR signaling pathway [10-12]. The phosphorylation of AMPK is essential for its biological function. Liver kinase B1 (LKB1) is the primary upstream regulator of AMPK through direct phosphorylation of AMPK [13-16]. However, the role of LKB1 in adult cardiomyocyte proliferation is unexplored. This study shows that the LKB1 protein level significantly increases after birth. Decreasing LKB1 expression promotes cardiomyocyte proliferation through the LKB1-AMPK-YAP axis, which may be a new target for adult cardiac regeneration after injury.

## MATERIALS AND METHODS

### Isolation and culture of neonatal rat ventricular myocytes

Neonatal cardiomyocytes were isolated by enzymatic disassociation of hearts from 1-2 days old Sprague–Dawley rats. The rats were euthanized with isoflurane (5%), after which the hearts were extracted. Hearts were, then, washed with ice-cold phosphate-buffered saline (PBS) and cut into pieces before enzymatic digestion using 1.25 mg/mL trypsin for 1 minute 3 times. The heart pieces were further digested using 0.8 mg/mL collagenase II for 30 min at 37°C. The collected digestion supernatant was added to an equal volume of Dulbecco’s modified Eagle’s medium (DMEM), supplemented with 10% fetal bovine serum (FBS), then filtered through a cellular strainer. Then, the cell suspension was incubated at 37°C for 90 min for differential attachment. Thereafter, the cells in the suspension were plated and cultured in DMEM with 10% FBS at 37°C in an atmosphere with 5% CO_2_ for 24 h. Then, siRNA (Synthesized by Ribobio company) was transfected into neonatal rat ventricular myocytes (NRVMs) using RNAiMAX (Invitrogen) for at least 24 h, and LKB1 overexpression vector (Synthesized by Sangon company) was transfected into NRVMs using lipofectamine 3000 transfection reagent (Invitrogen) for 48 h. Then, the cells were fixed with 4% polyformaldehyde for 20 min followed by immunostaining. Some cells were collected and stored at −80°C for subsequent analysis of protein and mRNA expression. The sequences of the siRNAs used in the experiments are listed in Supplemental Table S1.

### Immunostaining

The NRVMs were fixed with 4% paraformaldehyde for 10 min. The fixed cells and tissue sections were treated with Triton (0.1%) for 8 min and blocked with blocking buffer (Solarbio) for 30 min at 37°C. Then, the samples were incubated with the primary antibodies overnight at 4°C. After washing by PBS, the samples were incubated with fluorescent secondary antibodies for 1 h at 37°C, followed by 5 min of DAPI staining. After staining, microscopy was performed using Olympus confocal microscope (FluoView 1000). The primary antibodies used were anti-cardiac troponin T (#MA5-12960, Thermo Fisher Scientific), anti-α actinin (#A7811, Sigma), anti-Ki67 (#9027S, Cell-Signaling Technology), anti-pH3 (#PA5-17869, Thermo Fisher Scientific), and anti-Aurora B (#ab2254, Abcam). The secondary antibodies used were goat anti-mouse IgG (H+L), Alexa Fluor Plus 488 (#A-32723, Thermo Fisher Scientific), Goat anti-rabbit IgG (H+L), and Alexa Fluor Plus 488 (#A-11035, Thermo Fisher).

### RNA isolation, reverse transcription, and real-time qPCR

Total RNA was extracted using RNAiso Plus (#9109, Takara) from cell or tissue samples. For qRT-PCR of the genes, 2 mg RNA samples were reverse-transcribed to cDNA in a 20 ml reaction system, using RT master mix (RR036A, Takara). In each analysis, 1 ml cDNA was used for real-time qPCR. Bio-Rad software was used to calculate relative mRNA expression and normalized by *Gapdh*. All experiments were repeated at least three times. The primer sequences used are listed in Supplemental Table S1.

### Nuclear and protein extraction and western blotting

Cell nuclear and cytoplasmic proteins were extracted using a nuclear-cytoplasmic protein extraction kit (#P0028, Beyotime), following the manufacturer’s instructions. Cell and tissue proteins were extracted using RIPA buffer (#P0013B, Beyotime). Protein concentrations were measured with the enhanced BCA Protein Assay Kit (#P0012, Beyotime). SDS-PAGEs of the samples (30-50 μg) were conducted with 10–15% polyacrylamide gel. The electrophoresed proteins were electro-transferred into nitrocellulose filter membranes that were blocked with Tris-buffered saline, containing 5% non-fat milk. The membranes were incubated with primary antibodies overnight at 4°C and incubated with secondary antibodies at the room temperature for 1 h. The Odyssey Infrared Imaging System (Licor Biosciences) was used to visualize the bands, and Image J software was used to analyze the relative intensities of the protein bands. The antibodies used: anti-YAP (#14074, Cell Signaling Technology), anti-pYAP (#4911, Cell Signaling Technology), anti-AMPK (#5831, Cell Signaling Technology), anti-pAMPK (#bs-4002R, Bioss), anti-CCND (#55506, Cell Signaling Technology), anti-LKB1 (#3050, Cell Signaling Technology), anti-CDK4 (#bs-0633R, Bioss), and anti-CCNB (#bs-0572R, Bioss). Antibody information is presented in Supplemental Table S2.

### Statistical analysis

All data are expressed as means ± standard error of the mean. Statistical analysis was performed using GraphPad prism 8. Significant differences between and among groups were determined by one-way ANOVA for groups larger than 2 or Student’s t-test for groups of 2. *p* < 0.05 was considered significant.

## RESULTS

### LKB1 expression negatively correlates with neonatal cardiomyocyte proliferation in rodents

**FIGURE 1:**
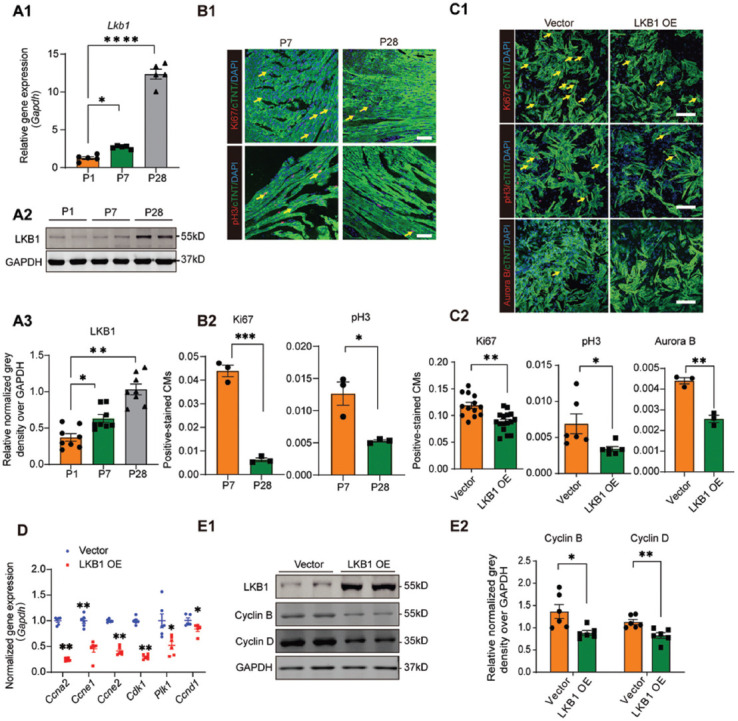
The increasing expression of LKB1 inhibits proliferation of NRVMs. (A) The relative mRNA and protein expressions of LKB1 in the heart at different postnatal time point are shown. Relative mRNA levels of *Lkb1* in P1, P7, and P28 mice hearts were assayed by qRT-PCR (n = 5) in A1; (A2) Relative protein expression of LKB1 in P1, P7, and P28 mice hearts were detected by western blots (n = 8); (A3) The quantitative grayscale density of western blots of A2; GAPDH was used for normalization; (B) Representative confocal microscopy of Ki67 and pH3 immunostaining at P7 and P28 hearts (Ki67^+^, pH3^+^ in red, and cTnT^+^ in green indicate proliferating cardiomyocytes). The Ki67 and pH3 positive cell percentages (from total >1000 cells) were calculated and shown in B2, scale bar = 50 μm. (C) Confocal microscopy of cell proliferation of NRVMs with LKB1 overexpression (Ki67^+^, pH3^+^, and Aurora B^+^ in red, and cTnT^+^ in green indicate proliferating cardiomyocytes), quantified in C2. 2000–5000 cells were randomly selected and quantified in each group, scale bar = 20 μm; (D) Cell cycle-associated gene expressions of NRVMs treated with vector or LKB1 overexpression plasmid were detected by qRT-PCR; (E) Representative images (E1) of cell cycle-related protein expression and quantification in E2, were analyzed by western blots (n = 6, GAPDH was used as internal control), *p < 0.05, **p < 0.01, ***p < 0.001. *p*: Postnatal day; NRVMs: Neonatal rat ventricular myocytes; LKB1: Liver kinase B1; GAPDH: Glyceraldehyde-3-phosphate dehydrogenase; qRT-PCR: Quantitative real-time PCR.

**FIGURE S1:**
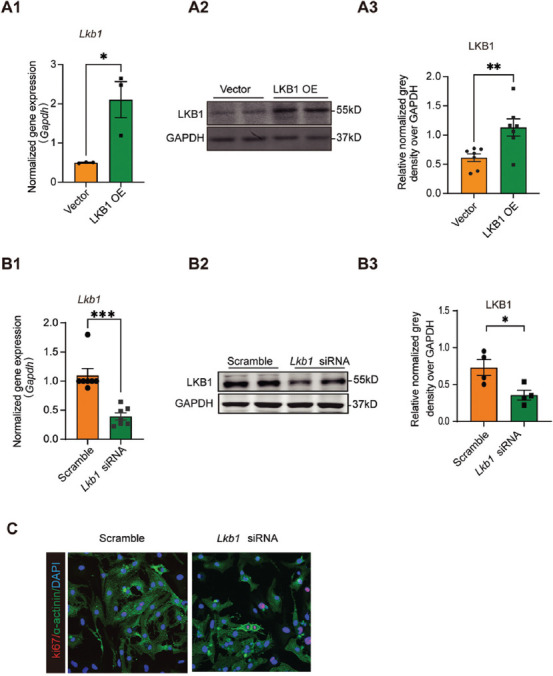
(A) The efficiency of LKB1 overexpression vector in NRVMs; (A1) qRT-PCR of *Lkb1* mRNA levels in NRVMs with LKB1 overexpression (n = 3); (A2) Western blots of NRVMs post-LKB1 overexpression (n = 7); (A3) The quantitative grayscale density of western blots of A2; (B) The efficiency of *Lkb1* siRNA in NRVMs; (B1) *Lkb1* mRNA levels in NRVMs transfected with scramble siRNA or *Lkb1* siRNA, measured by qRT-PCR (n = 7); (B2) Western blots of LKB1 proteins in NRVMs treated with scrambled siRNA or *Lkb1* siRNA (n = 4). Quantitative greyscale density for B3; *p < 0.05, **p < 0.01, ***p < 0.001; (C) The representative image of cytoskeleton depolymerization in *Lkb1* knockdown cardiomyocyte. NRVMs: Neonatal rat ventricular myocytes; AMPK: AMP-activated protein kinase; LKB1: Liver kinase B1.

To investigate the LKB1 protein expression in the postnatal heart, mouse heart tissues were collected at postnatal day 1 (p1), p7, and p28. Both real-time qPCR and western blotting consistently showed that LKB1 mRNA and protein levels increased from p1 to p28 (Figure 1A), and that cardiomyocyte proliferation from p7 to p28 decreased (Figure 1B), implying that LKB1 expression negatively correlates with the capability of cardiomyocyte proliferation. Next, the ectopic LKB1 overexpression construct was generated, and the increase in mRNA and protein levels after plasmid transfection were validated (Supplemental Figure S1A). To assess the effect of LKB1 on neonatal cardiomyocyte proliferation, ectopic LKB1 expression construct was transfected into rat neonatal cardiomyocytes, and the cell proliferation rate was assayed through Ki67, pH3, and Aurora B staining. All staining results showed a reduction in cardiomyocyte proliferation upon LKB1 overexpression (Figure 1C). In addition, the mRNA levels of cell cycle-related genes, including Cyclin A2 (*Ccna2*), Cyclin E1 (*Ccne1*), Cyclin E2 (*Ccne2*), Cyclin-dependent kinase 1 (*Cdk1*), Polo-like kinase 1 (*Plk1*), and Cyclin D1 (*Ccnd1*), after LKB1 overexpression, were significantly down-regulated (Figure 1D). Moreover, the protein levels of Cyclin B and Cyclin D were reduced with LKB1 overexpression (Figure 1E).

### Silencing of *Lkb1* upregulates rat neonatal cardiomyocyte proliferation *in vitro*

**FIGURE 2:**
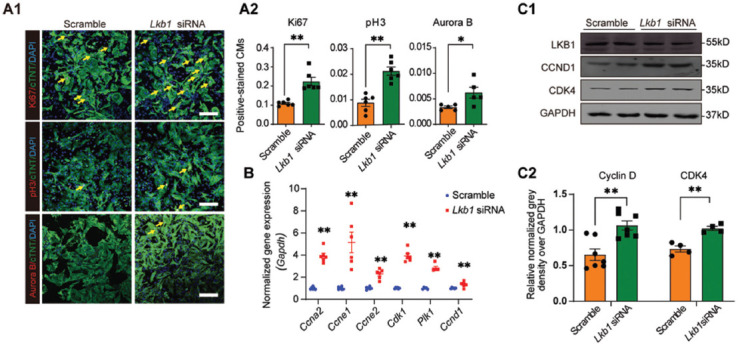
Knockdown of *Lkb1* upregulates NRVMs proliferation *in vitro*. (A) Cardiomyocyte proliferation was measured by immunofluorescence with Ki67, pH3, and Aurora B staining; (A1) Representative image of cell proliferation with Ki67, pH3, and Aurora B staining in NRVMs transfected with *Lkb1* siRNA; (A2) Quantitative results of Ki67, pH3, and Aurora B staining-positive cell percentage; 2000–5000 cells were randomly selected and quantified in each group, scale bar = 20 μm; (B) Real-time qPCR results for the mRNA levels of cell cycle-associated genes, including *Ccna2, Ccne1, Ccne2, Cdk1, Plk1, and Ccnd1*, in NRVMs transfected with scramble siRNA or *Lkb1* siRNA; (C) Western blots of proteins of cell cycle-associated genes; (C1) Representative images of western blots of cell cycle-related genes; the grayscale densitometry was quantified and summarized in C2. *p < 0.05, **p < 0.01, ***p < 0.001. NRVMs: neonatal rat ventricular myocytes; LKB1: liver kinase B1.

To perform loss-of-function study of *Lkb1*, siRNAs were transfected into neonatal cardiomyocytes, and real-time qPCR revealed approximately 60% of knockdown efficiency (Supplemental Figure S1B). Next, the cell proliferation assay with or without *Lkb1* knockdown was determined by Ki67, pH3, and Aurora B staining. All results showed that the loss of LKB1 significantly promotes NRVMs proliferation (Figure 2A). Moreover, we also noticed cytoskeleton (α-actinin) depolymerization in *Lkb1* knockdown cardiomyocyte (Supplemental Figure S1C). Less organized α-actin was found in NRVMs after *Lkb1* siRNA transfection, and Ki67 was co-stained to indicate the cell proliferation. In addition, the RNA levels of cell cycle-related genes, including *Ccna2, Ccne1, Ccne2, Cdk1, Plk1, and Ccnd1*, were determined by real-time qPCR, and Cyclin D, and CDK4 were determined by western blotting. Our data demonstrated that *Lkb1* knockdown upregulates the cell proliferation-related genes at both RNA and protein levels (Figure 2B and C).

### Knockdown of *Lkb1* promotes cardiomyocyte proliferation by enhancing the YAP signaling pathway

**FIGURE 3:**
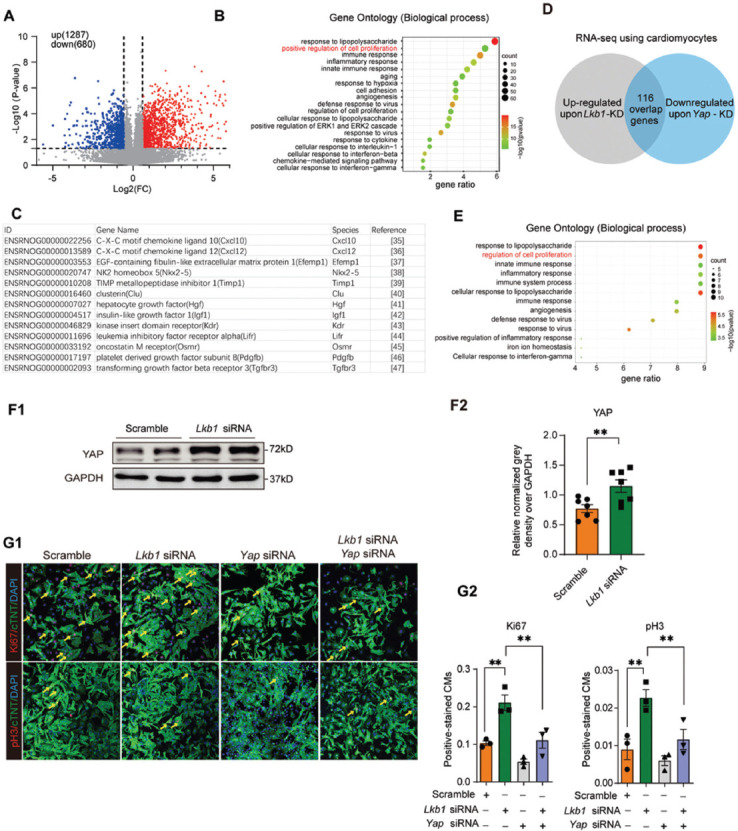
Knockdown of *Lkb1* promotes cardiomyocyte proliferation by activating YAP. (A) The volcano plot of bulk RNA-seq showing NRVMs transfected with *Lkb1* siRNA (n = 3); (B) The gene ontology analysis of upregulated genes in NRMVs with *Lkb1* silencing; (C) Genes which were associated with the YAP signaling pathway; (D-E) The Venn diagram revealed 116 overlap genes from the upregulated genes after *Lkb1* knockdown and downregulated genes after *Yap* silencing; the gene ontology analysis is shown in E; (F) Western blotting of YAP in NRVMs transfected with *Lkb1* siRNA or scramble control in F1; and the quantitative analysis of grayscale density in F2; (G) The confocal microscopy of Ki67- and pH3-positive cells in NRVMs transfected with *Lkb1* siRNA, *Yap* siRNA, combined *Lkb1* and *Yap* siRNA, and scramble control. The percentiles of Ki67^+^ or pH3^+^ cardiomyocytes are shown in G2 (more than 2000 cells were randomly selected and analyzed for each group) (n = 3). *p < 0.05, **p < 0.01, ***p < 0.001. NRVMs: Neonatal rat ventricular myocytes; LKB1: Liver kinase B1; YAP: Yes-associated protein

**FIGURE S2:**
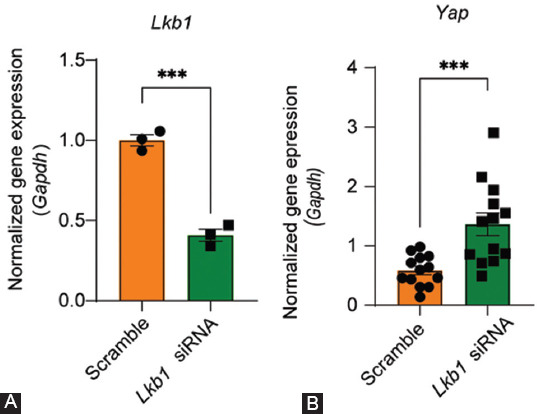
(A) Repressive efficiency of *Lkb1* siRNA was assayed by real-time qPCR in RNA-seq; (B) Relative mRNA levels of *Yap* in NRVMs treated with scramble or *Lkb1* siRNA, assessed by qRT-PCR. *p < 0.05, **p < 0.01, ***p < 0.001. NRVMs: Neonatal rat ventricular myocytes; YAP: Yes-associated protein.

To uncover the underlying mechanisms of *Lkb1* knockdown-induced cardiomyocyte proliferation, high throughput RNA-seq was performed using rat neonatal cardiomyocytes transfected with *Lkb1* siRNA versus scramble control. The repressive efficiency of *Lkb1* siRNA *in vitro* was assayed by real-time qPCR as shown (Supplemental Figure S2A). The RNA-seq data showed differentially expressed genes; 1,287 genes were upregulated (>1.5-fold change, transcripts/million [TPM]) and 680 genes were down-regulated (<0.67-fold change, TPM) (Figure 3A). Gene ontology analysis, using upregulated genes with *Lkb1* silencing, revealed that positive regulation of cell proliferation is one of the essential biological processes which align with our previous phenotypic findings (Figure 3B). Interestingly, in the gene set of the positive regulators of cell proliferation, 13 out of 25 genes were associated with the YAP/TAZ signaling pathway (Figure 3C), suggesting that YAP plays an important role in regulating cardiomyocyte proliferation upon *Lkb1* knockdown. We also combinatorially analyzed the upregulated genes with *Lkb1* silencing and down-regulated genes with *Yap* silencing [17] which revealed 116 genes co-regulated by *Lkb1* and *Yap* (Figure 3D). Analysis of the biological function of the co-regulated genes emphasized the regulation of cell proliferation (Figure 3E). Western blotting analysis demonstrated significant upregulation of YAP protein and mRNA upon *Lkb1* knockdown in NRVMs (Figure 3F, Supplemental Figure S2B).

To validate whether the stimulation of cardiomyocyte proliferation during LKB1 deficiency is mediated by the YAP signaling pathway, *Lkb1* and *Yap* siRNAs were co-transfected into neonatal cardiomyocytes, and Ki67 and pH3 staining was performed, which revealed that both Ki67 and pH3 positive cells were increased upon *Lkb1* knockdown. By contrast, *Yap* knockdown reversed *Lkb1* deficiency-induced cardiomyocyte proliferation, suggesting that YAP is an important downstream effector of LKB1 (Figure 3G).

### AMPK plays an essential role in LKB1 inhibition-induced cardiomyocyte proliferation

**FIGURE 4:**
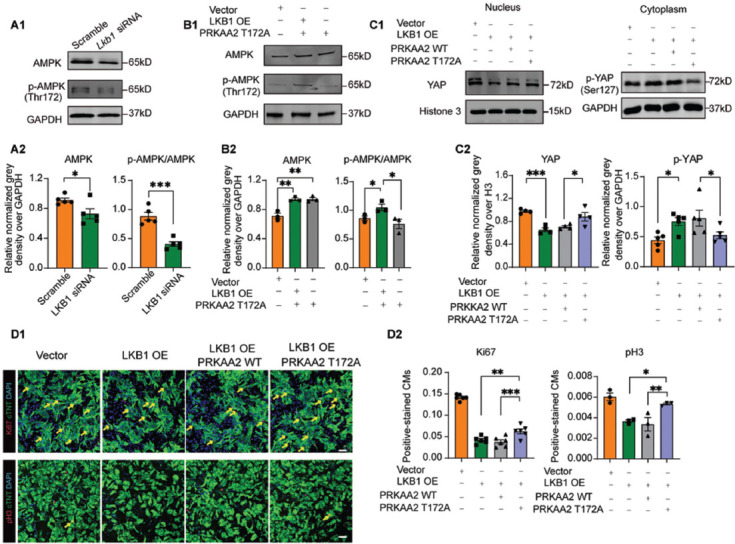
Role of AMPK on LKB1-mediated promotion of cardiomyocyte proliferation. (A) NRVMs were transfected with Lkb1 siRNA and scramble siRNA which served as a negative control. Representative western blots of AMPK, p-AMPK (T172) proteins are shown in A1 and quantitative analysis of greyscale density in A2; (B) NRVMs with LKB1 overexpression plasmid alone or with PRKAA2 T172A plasmid; vector plasmid-treated cells were used as a negative control. Representative western blots of AMPK and p-AMPK are shown in B1, and the quantitative analysis of grayscale density for B2; (C) Representative western blots of YAP and p-YAP in nucleus and cytoplasm in NRVMs treated with LKB1 overexpression plasmid alone, PRKAA2 wild-type (WT) plasmid, or PRKAA2 T172A plasmid. Anti-Histone 3 and anti-GAPDH antibodies were used as the nucleus- and cytoplasm-specific control. The quantitative analysis of grayscale density is shown in C2; (D1) Confocal microscopy of cell proliferation markers Ki67 and pH3 in NRVMs treated with LKB1 overexpression alone, PRKAA2 WT plasmid, or PRKAA2 T172A plasmid; (D2) The quantitative summaries of Ki67^+^/pH3^+^ cell percentages are shown. Scale bar = 20 μm. *p < 0.05, **p < 0.01. ***p< 0.001. NRVMs: Neonatal rat ventricular myocytes; AMPK: AMP-activated protein kinase; LKB1: Liver kinase B1; GAPDH: Glyceraldehyde-3-phosphate dehydrogenase; YAP: Yes-associated protein.

**FIGURE S3:**
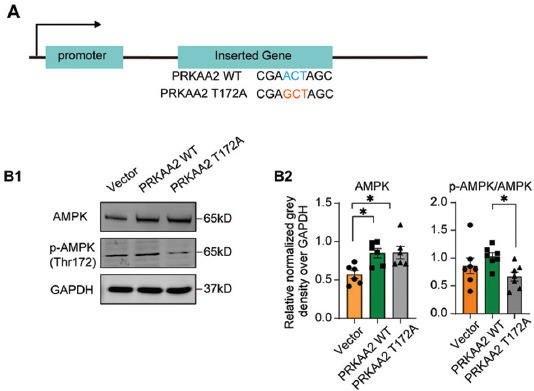
(A) Schematic diagram of plasmid construction; (B) NRVMs have been transfected with PRKAA2WT plasmid or PRKAA2 T172A plasmid or vector plasmid which served as a negative control. Representative western blotting images of AMPK and p-AMPK are shown in B1 and the quantitative analysis of grayscale density in B2. NRVMs: Neonatal rat ventricular myocytes; AMPK: AMP-activated protein kinase.

AMPK is an essential and well-known downstream substrate of LKB1, and the phosphorylation of AMPK by LKB1 confers AMPK’s biological activity [14]. In the above context, we found that YAP is a downstream effector of LKB1. Therefore, we were curious whether AMPK mediates the YAP signal activation upon *Lkb1* knockdown in cardiomyocytes [18]. We found that *Lkb1* siRNA significantly repressed AMPK phosphorylation, as well as AMPK protein expression (Figure 4A). To clarify the role of AMPK phosphorylation on LKB1 deficiency-mediated cardiomyocyte proliferation, we generated AMPK wild-type (PRKAA2-WT) and AMPK mutant construct (PRKAA2-T172A) that deprived phosphorylation activity by replacing threonine to alanine at 172 amino acid (Supplemental Figure S3A). Both constructs were transfected into cardiomyocytes, and the western blotting data revealed that overexpression of PRKAA2-WT increased total and phospho-AMPK, whereas PRKAA2-T172A increased total AMPK protein but prevented T172A phosphorylation of AMPK, indicating the PRKAA2-T172A inhibited endogenous AMPK phosphorylation (Supplemental Figure S3B). We then co-transfected PRKAA2-T172A with LKB1 overexpression construct in cardiomyocytes and found that PRKAA2-T172A completely blocked the LKB1-mediated AMPK phosphorylation (Figure 4B). More importantly, our results showed that transfection of PRKAA2-T172A effectively blocked the LKB1 overexpression-mediated downregulation of YAP protein in the nucleus and upregulation of YAP phosphorylation in the cytoplasm in cardiomyocytes (Figure 4C). Furthermore, PRKAA2-T172A partially reversed the LKB1-mediated downregulation of Ki67 and completely reversed the LKB1-mediated downregulation of pH3, showing its anti-proliferative effect in cardiomyocytes (Figure 4D). Together, the above data demonstrated that AMPK phosphorylation plays an essential role in the LKB1-YAP regulatory pathway.

## DISCUSSION

The stimulation of cardiomyocyte regeneration post-cardiac injury is very challenging because cardiomyocytes quit the cell cycle shortly after birth, with no significant increase in cell number [19]. Different from other cell types, cardiomyocytes undergo a fundamental metabolic switch before and after birth; the primary energy resource of prenatal cardiomyocytes is provided by glycolysis, whereas postnatal cardiomyocytes rapidly shift to fatty acids oxidation as the primary energy source within days after birth [20]. Although many studies have investigated the importance of energy transition in cardiomyocytes [21], the mechanisms that lead to adult cardiomyocyte proliferation are unclear. Our study revealed that the LKB1 expression level in cardiomyocytes is upregulated after birth and that LKB1 abundance negatively correlated with neonatal rat cardiomyocyte proliferation through loss- and gain-of-function study *in vitro*. Our mechanism study uncovered that LKB1 negatively regulates cardiomyocyte proliferation through the LKB1-AMPK-YAP signal pathway.

AMPK, as a critical energy monitor, senses the changes of AMP/ATP ratio in the cytoplasm [9]. The activation of AMPK inhibits anabolic metabolism and enhances catabolic processes, including that induced by β-oxidation. AMPK is composed of three heterotrimeric complexes: α, β, and γ subunit, among which the α subunit is catalytic subunit, and β and γ are the regulatory subunits [22]. The phosphorylation of threonine 172 (T172) in the α subunit plays an essential role in the functioning of AMPK [23]. In the heart, AMPK is activated by two AMPK kinases: LKB1 and CaMKKβ [24]. The serine/threonine kinase LKB1 responds to anoxic stress and phosphorylates the T172 residue of the AMPK’s α subunit [25]. Our results indicated that the LKB1-induced AMPK phosphorylation (T172) subsequently enhanced the phosphorylation of YAP and inactivated the biological function of YAP. YAP is the nuclear target of the Hippo signaling pathway, which stimulates cardiomyocyte proliferation but not hypertrophy [26]. We showed that knockdown of LKB1 suppressed the phosphorylation of AMPK and activated YAP, which subsequently promoted cardiomyocyte proliferation. Furthermore, our results also suggested novel crosstalk between LKB1-AMPK and Hippo signaling pathway in regulating cardiomyocyte proliferation through YAP.

Adult cardiomyocyte proliferation, in general, is extremely low [27]. In the early postnatal period, cardiomyocytes undergo limited proliferation after injury [28-30]. The significant obstacles of cardiomyocyte proliferation include: (1) stalled cell cycle, (2) specialized cytoskeleton, and (3) switched metabolic pathway [5,31,32]. Interestingly, AMPK is involved in all these three processes: AMPK senses energy stress and enhances catabolic metabolism [33]; AMPK mediates re-organization of the cytoskeleton [34], and AMPK inhibits cell proliferation [35]. Therefore, AMPK is one of the most critical elements in cardiomyocyte proliferation. However, direct repression of AMPK is very challenging because AMPK is composed of 12 different proteins which are categorized into 3 subunits [13]. Therefore, it is more feasible to regulate AMPK activity by targeting an upstream regulator rather than the heterotrimeric complex, per se. Our study implied a therapeutic target for stimulating cardiomyocyte proliferation post-injury by knocking down LKB1 expression.

## CONCLUSION

Our study uncovered *Lkb1* knockdown as a new intervention target for inducing postnatal cardiomyocytes to re-enter the cell cycle. Our findings may provide an alternative approach to heart regeneration.

## Figures and Tables

**TABLE S1:**
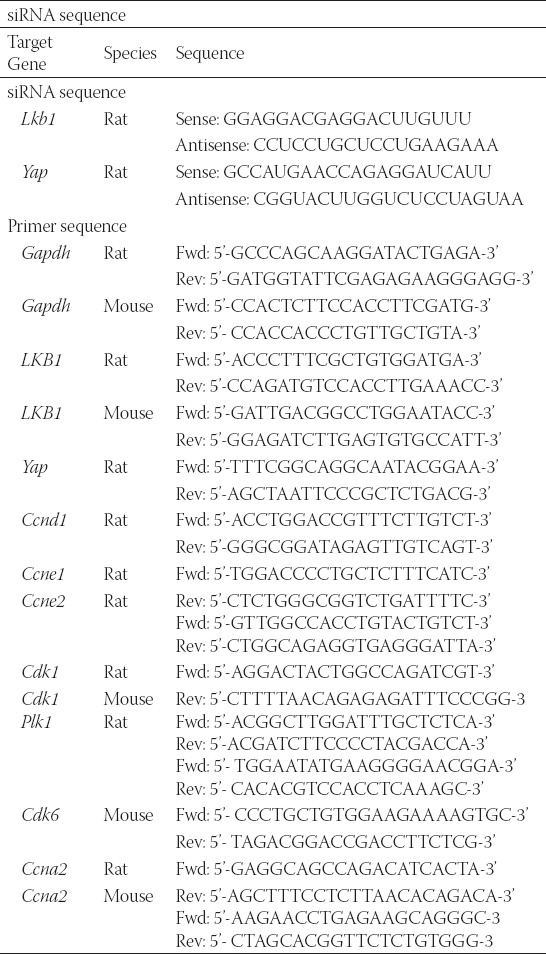
siRNA and primers used in the study

**TABLE S2:**
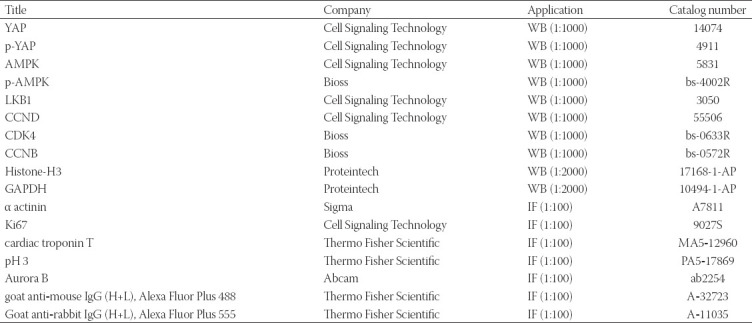
Antibody information
