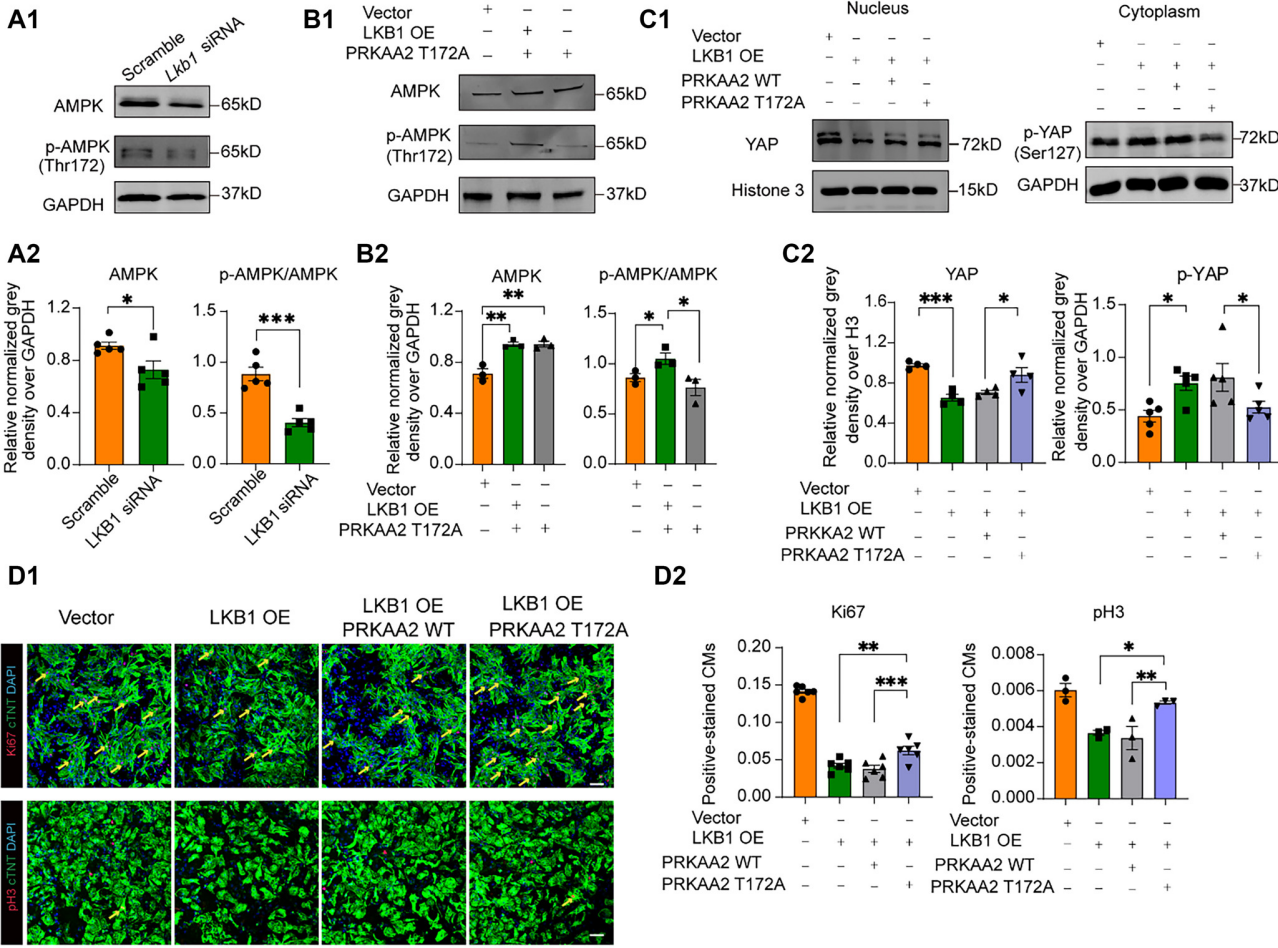# Correction: LKB1 suppression promotes cardiomyocyte regeneration via LKB1-AMPK-YAP axis

**DOI:** 10.17305/bb.2025.12567

**Published:** 2025-04-25



Shuang Qu, Qiao Liao, Cheng Yu, Yue Chen, Hao Luo, Xuewei Xia, Duofen He, Zaicheng Xu, Pedro A. Jose, Zhuxin Li, Wei Eric Wang, Qing Rex Lyu, Chunyu Zeng

Vol. 22 No. 5 (2022) | https://doi.org/10.17305/bjbms.2021.7225 | 29 April, 2022

In the published article [Qu S, et al. LKB1 suppression promotes cardiomyocyte regeneration via LKB1-AMPK-YAP axis. Bosn J Basic Med Sci. 2022; DOI: 10.17305/bjbms.2021.7225], an error was identified in Figure 4D1. Specifically, the immunofluorescence image originally belonging to the “vector” group was mistakenly used for the “LKB1 OE” group during figure preparation. This was an inadvertent error in image placement.

The corrected version of Figure 4 is shown below. This correction does not affect the statistical analysis, interpretation, or the overall conclusions of the study.